# Insect cells are superior to *Escherichia coli *in producing malaria proteins inducing IgG targeting PfEMP1 on infected erythrocytes

**DOI:** 10.1186/1475-2875-9-325

**Published:** 2010-11-15

**Authors:** Michala E Victor, Anja Bengtsson, Gorm Andersen, Dominique Bengtsson, John P Lusingu, Lasse S Vestergaard, David E Arnot, Thor G Theander, Louise Joergensen, Anja TR Jensen

**Affiliations:** 1 Centre for Medical Parasitology, Department of International Health, Immunology and Microbiology, Faculty of Health Sciences, University of Copenhagen and Department of Infectious Diseases, Copenhagen University Hospital (Rigshospitalet), CSS Oester Farimagsgade 5, Building 22 & 23, PO 2099, 1014 Copenhagen K, Denmark; 2 National Institute for Medical Research, Tanga Centre, Tanga, Tanzania; 3 Institute of Infection and Immunology Research, School of Biology, University of Edinburgh, West Mains Road, Edinburgh, EH9 3JT, Scotland, UK

## Abstract

**Background:**

The PFD1235w *Plasmodium falciparum *erythrocyte membrane protein 1 (PfEMP1) antigen is associated with severe malaria in children and can be expressed on the surface of infected erythrocytes (IE) adhering to ICAM1. However, the exact three-dimensional structure of this PfEMP1 and its surface-exposed epitopes are unknown. An insect cell and *Escherichia coli *based system was used to express single and double domains encoded by the *pfd1235w var *gene. The resulting recombinant proteins have been evaluated for yield and purity and their ability to induce rat antibodies, which react with the native PFD1235w PfEMP1 antigen expressed on 3D7_PFD1235w_-IE. Their recognition by human anti-malaria antibodies from previously infected Tanzanian donors was also analysed.

**Methods:**

The recombinant proteins were run on SDS-PAGE and Western blots for quantification and size estimation. Insect cell and *E. coli*-produced recombinant proteins were coupled to a bead-based Luminex assay to measure the plasma antibody reactivity of 180 samples collected from Tanzanian individuals. The recombinant proteins used for immunization of rats and antisera were also tested by flow cytometry for their ability to surface label 3D7_PFD1235w_-IE.

**Results:**

All seven pAcGP67A constructs were successfully expressed as recombinant protein in baculovirus-infected insect cells and subsequently produced to a purity of 60-97% and a yield of 2-15 mg/L. By comparison, only three of seven pET101/D-TOPO constructs expressed in the *E. coli *system could be produced at all with purity and yield ranging from 3-95% and 6-11 mg/L. All seven insect cell, but only two of the *E. coli *produced proteins induced antibodies reactive with native PFD1235w expressed on 3D7_PFD1235w_-IE. The recombinant proteins were recognized in an age- and transmission intensity-dependent manner by antibodies from 180 Tanzanian individuals in a bead-based Luminex assay.

**Conclusions:**

The baculovirus based insect cell system was distinctly superior to the *E. coli *expression system in producing a larger number of different recombinant PFD1235w protein domains and these were significantly easier to purify at a useful yield. However, proteins produced in both systems were able to induce antibodies in rats, which can recognize the native PFD1235w on the surface of IE.

## Background

Malaria remains a devastating infectious disease, with the parasite *Plasmodium falciparum *being responsible for killing approximately one million children below the age of five each year [1]. Development of an effective malaria vaccine would have a profound impact on the control of the disease as drug resistance toward affordable, effective drugs continues to emerge. However, since nearly 60% of hypothetical proteins in the parasite genome are of unknown function, much needs to be learned about the biology of the parasite. In particular, identifying parasite antigens that elicit long-lasting protective immune response has proven very difficult [2].

In the discovery and characterization of new vaccine candidates, bioinformatics tools are useful, but protein function and structure cannot be definitely determined from exclusively *in silico *experiments. However, sufficient quantities of the "protein of interest" cannot usually be isolated from either host infection or *in vitro *culture host. Therefore, heterologous expression of soluble and functional parasite proteins is key to progress toward identification of new vaccine candidates.

The classic genetic model bacterium, *Escherichia coli*, is still a preferred organism for heterologous expression of recombinant proteins, largely due to cost considerations, speed, ease of use and genetic manipulation. But for many proteins the bacterial cell often does not produce satisfactory results [3]. Obstacles to the efficient expression of *Plasmodium *proteins in bacteria are their usually high molecular weight (> 56 kDa), more basic pI (> 6), lack of homology to bacterial proteins, *Plasmodium*-specific inserts, often apparently disordered sequences, transmembrane regions, signal peptides, disulphide bridges and export motifs [4-6]. In addition to *E. coli*, several other organisms have, therefore, been employed to attempt to improve heterologous expression, including baculovirus-infected insect cells [7]. This system has the advantage of usually permitting production of high molecular weight recombinant proteins, with recognition of normal eukaryotic targeting signals and post-translational machinery. However, the baculovirus expression system requires an increased investment time and uses higher cost media compared to bacteria, and it is technically more challenging [3].

Producing recombinant proteins retaining natural folding is essential for elucidating the three dimensional structure of malaria proteins and for determining which structural epitopes are exposed on the surface of IE during natural malaria infections. Both bacteria and insect cells have been shown to express *Plasmodium *proteins, which retained conformational epitopes and elicited antibody responses [8-10], and both systems have produced recombinant merozoite surface protein 1 (MSP1) capable of acting as a protective immunogen and capable of protecting mice and monkeys from malaria challenge [11,12]. Existing data thus do not indicate that one heterologous expression system is definitely superior to the other and although bioinformatics tools can predict solubility and aid the design of suitable domains for heterologous expression, such predictions are not powerful and the identification of the optimal system for heterologous expression remains a matter of experimental testing.

The *P. falciparum *erythrocyte membrane protein 1 (PfEMP1) proteins are implicated in two key biological phenomena in malaria; antigenic variation and sequestration [13,14], and have, therefore, been proposed as candidates for a blood stage vaccine [15]. The PfEMP1 proteins are encoded by the *var *gene family, with approximately 60 genes per haploid genome. Each gene consists of a highly variable 5' exon encoding an extracellular region and a single transmembrane domain, a relatively conserved intron, and a highly conserved 3' exon encoding an intracellular region of the transmembrane protein. The *var *genes vary in size from 6 - 15 kb and encode PfEMP1 proteins ranging in size from 200 - 500 kDa, the variable extracellular region being composed of several distinct domain structures; the N-terminal segment (NTS, 75-100 aa), Duffy-binding like domains (DBL, α-ε, 100-250 aa) and the cysteine-rich interdomain (CIDR, α-γ, 70-200 aa) [2,16]. A few higher order arrangements of the domains are conserved, such as the NTS-DBL1α-CIDR1α combination always found at the amino terminus (referred to as the head structure) and the DBLδ-CIDRβ/γ tandem pairing [17,18].

The high molecular weight of the full-length PfEMP1 proteins complicates full-length heterologous expression, and the PfEMP1 proteins are usually expressed as single or double domains. However, even though the size of single domains are compatible with most expression systems, they still encompass biochemical and physical characteristics, such as a high number of disulphide bridges and a basic pI (> 6), that, in theory, make recombinant expression difficult.

In previous studies [19,20], the parasite expression of the PfEMP1 protein encoded by the *var pfd1235w *gene, was found to be associated with severe childhood malaria. However, the exact role of the PFD1235w protein during childhood malaria infections, including definition of the domain/motif binding host endothelium ICAM1 and identification of immunodominant epitopes remains to be established. Such studies require an expression system producing experimentally useful quantities of recombinant PFD1235w protein, with a native fold. To address this issue, an *E. coli *and baculovirus expression system was used to express single and double domains from the extracellular region of the PFD1235w protein. The resulting proteins were evaluated in terms of yield and purity, ability to induce antibodies reactive with native PFD1235w expressed on the surface of IE and their recognition by Tanzanian immune plasma.

## Methods

### Protein expression

The 3D7 DNA sequences encoding the PFD1235w protein domains listed in Additional file 1 was PCR amplified, cloned and expressed in a baculovirus insect cell system as described [19,21,22]. Supernatants with secreted recombinant protein were diafiltered on an ÄKTAcrossflow (GE Healthcare) using Buffer A (20 mM sodium phosphate, 500 mM sodium chloride, pH 7.5) plus 10 mM imidazole prior to purification on a HIS-select Cartridge column (Sigma-Aldrich). Bound recombinant protein was eluted using a one-step procedure and Buffer A including 200 mM imidazole. Following dialysis against buffer A the final protein concentration was measured at UV_280 nm_.

Similarly, 3D7 *pfd1235w *DNA sequences (Additional file 1) were PCR amplified and cloned in the *E. coli *pET101/D-TOPO vector by blunt-end ligation, recombinant plasmid transfected into the Rosetta-gami B (DE3) *E. coli *strain with improved cytoplasmic disulphide bond formation (Novagen) and protein expressed in 2x YT media (1.6% (w/v) Bacto tryptone, 1% (w/v) yeast extract, 0.5% NaCl, pH 7.5) using the Champion™ pET Directional TOPO^® ^Expression Kit as described (Invitrogen).

In brief, following dilution (1:100) of overnight cultures cells were grown to OD_600_= 1 at 16-21°C and induced with 10-1000 μM IPTG for 20 hours. Prior to large scale protein production the optimal IPTG concentration and temperature for induction of protein expression from each construct was determined in a pilot experiment using 10, 100, 1000 μM IPTG and incubating at 37°C for 3 hours, room temperature for 20 hours, and 4°C for 20 hours. The different IPTG concentrations only yielded minor differences in expression levels and in most cases 10 μM was used for large scale protein production. Similarly, varying the temperature only had a slight impact on expression levels with incubation at room temperature for 20 hours giving the highest yield. Following harvesting, cell pellets were resuspended in 20 ml Buffer A plus 10 mM Imidazole and a Complete Mini Protease Inhibitor Tablet without EDTA (Roche). Following sonication, centrifugation and filtration supernatants were purified on a HISTrapHP column (GE Healthcare). Elution, dialysis and estimation of protein concentration was carried out as described above.

### SDS-PAGE and Western blotting

For purity and quality estimation 2 μg of each purified recombinant protein, both reduced (+DTT) and non-reduced (- DTT), was separated on a NuPAGE^® ^Novex 4-12% Bis-Tris gel in MOPS SDS Running buffer (Invitrogen) and detected using BioSafe coomassie stain (BIO-RAD). ProSieve Color Protein marker (Lonza) was used for calibration and Quantity One software (BIO-RAD) for protein quantification and size estimation. Western blotting was done using standard techniques. In brief, 0.2 μg purified protein was separated, blotted onto a Hybond™-C Extra membrane (Amersham BioSciences), and detected by an anti-V5-HRP antibody (Invitrogen) using a Chemiluminescent detection kit (Pierce).

### Plasma samples

The plasma samples were obtained during a cross-sectional survey in April 2001 as part of a longitudinal study characterizing malaria morbidity and immunity among individuals living in Mgome, Ubiri, and Magamba village in the Tanga region of Northeastern Tanzania. The point prevalence of *P. falciparum *in the three different villages was 81% (Mgome), 41% (Ubiri), and 12% (Magamba) [23]. Prior to this study, informed consent was obtained from all studied individuals and/or their parents and ethical clearance was granted by the Ministry of Health and the Ethics Committee (National Institute for Medical Research, Tanzania). For this study, 15 samples from each of four predefined age groups (2-4, 5-9, 10-14, and 15-19 years) were randomly picked and used for Luminex plex analysis of antibody reactivity to purified recombinant insect cell and *E. coli *produced proteins. All plasma samples represented individuals with a previous history of malaria, but no clinical symptoms at the time of sampling. Samples from 20 Danish donors with no previous exposure to malaria were included as controls.

### Luminex

The antibody reactivity of human plasma samples were tested in the BioPlex^100 ^System (BioRad) as previously described [24]. Briefly, 50 μg of six insect cell (DBL1α-CIDR1α, CIDR1α, DBL3β, DBL4γ, DBL5δ, DBL5δ-CIDR2β) and two *E. coli *(DBL3β, DBL4γ) produced proteins were individually coupled to 6.25 × 10^6 ^Luminex xMAP technology microsphere beads (Ramcon). Diluents used were PBS/TB (PBS, 0.05% (v/v) Tween-20, 0.1% (w/v) BSA, pH 7.4). Prior to multiplexing, protein coupling was verified by incubating 1250 coupled beads with 1:100 to 1:1000,000 mouse anti-V5 antibody (Invitrogen) followed by biotinylated anti-mouse IgG (1:1000, DakoCytomation). The biotinylated antibody was detected using PE-streptavidin (1:1000, Invitrogen). Multiplexed beads (1.88 × 10^4 ^per protein per well) were incubated with 180 different individual Tanzanian plasma samples (1:1300) followed by PE-conjugated anti-human IgG (1:3200, Jackson Immunoresearch). Beads were resuspended in 100 μl diluents and a minimum of 100 beads from each set of multiplexed beads were analyzed to yield the mean fluorescence intensity (MFI). To test for inter-plate variation, each plate included a pool of positive plasma titrated 2-fold from 1:40 to 1:20,480 in addition to a negative buffer control. Antibody reactivity of individual plasma samples was calculated as MFI_sample _minus MFI_buffer_. For each of the proteins the PBS buffer corrected mean MFI value plus three standard deviations obtained with plasma from 20 Danish blood donors without previous exposure to malaria was used as a negative cut-off value.

### Generation of antisera

All procedures complied with European or national regulations. Prior to immunization each animal was pre-bled and these sera were used as negative controls. Polyclonal rat antisera (2-3 rats per protein) were raised by subcutaneous injection of 25 μg recombinant protein in complete Freund's adjuvant followed by several boosters of 15 μg protein in incomplete Freund's adjuvant. Additional bleeds were collected during and following the completion of the immunization scheme.

### Blocking of antibodies

Rat PFD1235w DBL1α-CIDR1α, DBL3β, DBL4γ antisera binding was blocked by incubation for 1 hr at 4°C using excess purified recombinant homologous or heterologous produced protein (100 μl of 0.1 mg/ml protein in each well containing 5 μl rat serum) and remaining antibody reactivity to native PFD1235w expressed on the surface of 3D7_PFD1235w _infected erythrocytes was subsequently tested by flow cytometry as described below. The resulting reduction in serum reactivity was calculated as ((MFI_sample _- MFI_min_)/MFI_max_)*100% with MFI_min _being the signal obtained with addition of secondary antibody, but no antiserum and MFI_max _the signal obtained with addition of serum and secondary antibody, but no blocking protein prior to testing by flow cytometry.

### Malaria parasite cultivation and *in vitro *selection procedure

The *P. falciparum *3D7 clone was cultured in blood group 0 erythrocytes as previously described [25]. Cultures were routinely genotyped by PCR using primers targeting the polymorphic loci MSP2 and GLURP as described [26], and mycoplasma tested using the MycoAlert^® ^Mycoplasma Detection Kit (Lonza) following the manufactures instructions. IgG from rabbits immunized with DBL4γ of 3D7 PFD1235w expressed in insect cells were used to *in vitro *select a previously selected 3D7 line [27]. Briefly, the rabbit sera were depleted on human uninfected erythrocytes type 0, incubated with gelatine purified trophozoite-stage 3D7 parasites for 30 min at 37°C, and unbound antibodies were removed by washing. Subsequently, IE were incubated with Protein A-coupled Dynabeads^® ^(Invitrogen) for 30 min at 37°C and bound IE were trapped using a magnet. Trapped IE were transferred to new culture flasks for continued *in vitro *culturing and the procedure was repeated until cultures stained positive by the selecting antisera in flow cytometry.

### Flow cytometry

Flow cytometry surface staining was done with minor modifications as described in Staalsoe et al., 1999 [25]. Briefly, IE were purified on a magnet-activated cell sorting column (MACS), adjusted to 2 × 10^6 ^cells/ml and 100 μl cells were added to each well of 96 well plates and incubated for 30 min at 4°C with 5 μl rat sera depleted of anti-human erythrocyte antibodies. Following, IEs were incubated for 30 min at 4°C with 100 μl/well FITC-conjugated goat anti-rat IgG (Zymed) diluted 1:150 in PBS supplemented with 2% foetal calve serum and ethidium bromide added to a final concentration of 2 μg/ml. IEs were analysed on a Cytomics FC 500 MPL flow cytometer (Beckman Coulter), and data analysed using WinList version 6.0 (Verity Software House Inc.). IEs were gated based on the ethidium bromide staining to exclude non-infected erythrocytes. Mean fluorescent intensity (MFI) for individual rat bleeds were related to MFI for the corresponding pre-bleed sample (MFI bleed/MFI pre-bleed), and the sample with the highest relative MFI value for each animal or each immunization group were used for further analysis. A relative MFI value of 1.3 was used as cut-off for discriminating between positive and negative serum samples

### Confocal microscopy

Laser scanning confocal microscopy was performed on samples analysed by flow cytometry in order to observe the staining patterns on individual IE. MACS purified unlabelled infected cells from the same batches of parasites tested by flow cytometry were incubated with individual antibodies as previously described [28]. Briefly, 1 μl packed IE were washed in three times in 100 μl 1% BSA in PBS (BSA/PBS) and the pellet was incubated in 100 μl BSA/PBS and 3 μl of the respective antibodies for 30 minutes at 4°C. The IE were washed three times in BSA/PBS. The IE stained with primary antibody were then incubated with 100 μl secondary antibody Alexa 488^® ^anti-rat IgG (Invitrogen) and DAPI (3 μl of a 300 ng/ml solution) for 30 minutes at 4°C. The IE were washed three times in 100 μl 1% BSA/PBS and visualized as live, unfixed cells using a Nikon TE 2000-E confocal Nikon microscope with 60× oil immersion objective lens (DIC). The images were processed using Adobe Photoshop software and displayed with the 5 μm scale bar calculated by the EZ-C1 software.

## Results

### Yield and purity of recombinant PFD1235w protein

**Figure 1:**
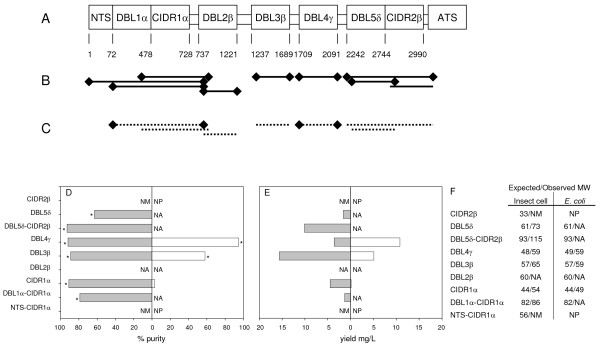
**The structure and characteristics of PFD1235w**. (A) Domain structure of PFD1235w with amino acid domain boundaries indicated. Recombinant PFD1235w protein domains produced in (B) baculovirus-infected insect cells (solid lines) and (C) *E. coli *(dotted lines). (D) Purity (%), (E) yield (mg/ml), (F) table of theoretical and observed molecular weight in kDa of insect cell and *E. coli *produced domains. Lines ending with a diamond in (B) and (C) indicates recombinant protein inducing IE surface reactive antibodies in rats and (*) constructs tested by Luminex. NM: not measured, NA: not applicable, NP: not produced.

**Figure 2:**
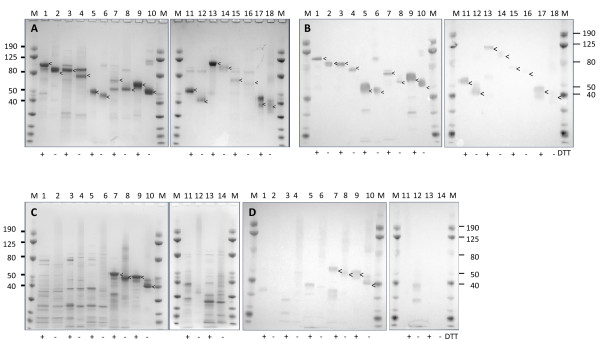
**Purity of recombinant PFD1235w protein domains expressed in insect cells and *E. coli***. (A and C) Sodium dodecyl sulfate-polyacrylamide (SDS-PAGE) gel electrophoresis of 2 μg/lane and (B and D) Western blotting of 0.2 μg/lane recombinant protein. (A and B) Insect cell produced protein (1-2) NTS-CIDR1α, (3-4) DBL1α-CIDR1α, (5-6) CIDR1α, (7-8) DBL2β, (9-10) DBL3β, (11-12) DBL4γ, (13-14) DBL5δ-CIDR2β, (15-16) DBL5δ, (17-18) CIDR2β and (C and D) *E. coli *produced (1-2) DBL1α-CIDR1α, (3-4) CIDR1α, (5-6) DBL2β, (7-8) DBL3β, (9-10) DBL4γ, (11-12) DBL5δ-CIDR2β, (13-14) DBL5δ. M: ProSieve Color Protein marker (Lonza). Samples were reduced using DTT (+) or non-reduced (-). Arrows indicate identified protein band.

Seven constructs encoding single or double domains from the extra-cellular region of the PfEMP1 protein encoded by *pfd1235w *were made for experimental comparative expression in both insect cells and an *E. coli *system. Successful expression and purification was achieved with all seven constructs in baculovirus-infected *Trichoplusia ni *High-5 cells (DBL1α-CIDR1α, CIDR1α, DBL2β, DBL3β, DBL4γ, DBL5δ-CIDR2β, DBL5δ). Three out of seven constructs (CIDR1α, DBL3β, DBL4γ) produced detectable recombinant protein in the Rosetta-gami B (DE3) *E. coli *strain, which has improved cytoplasmic disulphide bond formation (Figure 1). All recombinant proteins were purified and checked by sodium dodecyl sulfate-polyacrylamide gel electrophoresis and Western blotting as described previously (Figure 2) [19].

Five insect cell-derived proteins were processed to a purity greater than or equal to 80%, whereas similar purity was only achieved with one bacterial expressed domain, DBL4γ (Figure 1). The highest protein yield observed was achieved with the DBL3β in insect cells (app. 15 mg/L) with *E. coli *yielding a maximum of 11 mg/L for the DBL4γ domain. With regards to estimating the molecular weight of the products, in comparison with the calculated expected molecular weight a 20% average increase was observed when expressing protein domains in insect cells. This was opposed to an average of 12% increase observed in the *E. coli *expression system (Figure 2). This 8% difference in size is explained by glycosylation of the secreted recombinant protein expressed by the insect cells. Degradation of the PFD1235w domains was more pronounced in the *E. coli *expression system with only DBL4γ showing no sign of degradation (Figure 2). By contrast, DBL1α-CIDR1α, DBL4γ, DBL5δ-CIDR2β, and DBL5δ expressed in the baculovirus system were purified with no detectable degradation (Figure 2). In addition to this, three additional proteins DBL1α, NTS-CIDR1α, and CIDR2β were attempted expressed in insect cells, but not in *E. coli*. Of these proteins only DBL1α failed to express.

### Recombinant PFD1235w protein induces IE surface reactive antibodies following immunization

Identification of surface-exposed PfEMP1 domains accessible to antibodies has often been hampered by difficulties in generating specific surface-reactive anti-PfEMP1 antibodies when using recombinant antigen as the immunogen. To address this issue for the particular PfEMP1 of interest, that associated with severe malaria in children [19], rats were immunized with recombinant PFD1235w protein domains expressed in insect cells (NTS-CIDR1α, DBL1α-CIDR1α, CIDR1α, DBL2β, DBL3β, DBL4γ, DBL5δ-CIDR2β, DBL5δ, CIDR2β) or *E. coli *(DBL1α-CIDR1α, CIDR1α, DBL3β, DBL4γ).

**Figure 3:**
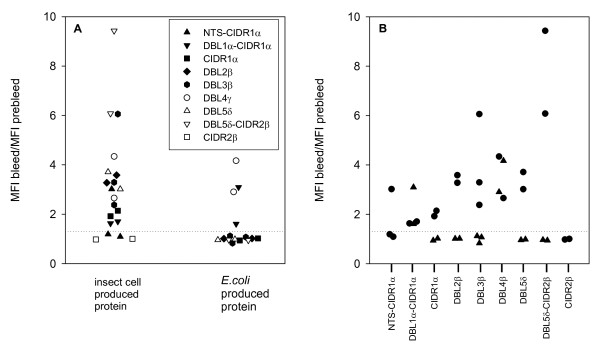
**IE surface reactivity of sera raised against insect cell and *E. coli *produced domains of PFD1235w**. (A) IE surface reactivity of rat antiserum raised against recombinant PFD1235w as indicated in the legend and measured by flow cytometry. (B) Antibodies against C-terminal located PFD1235w domains show higher IE surface reactivity than antibodies against N-terminal domains. Circles and triangles in (B) show antibody reactivity of plasma from rats immunized with insect cell and *E. coli *produced domains, respectively. Two-three rats were immunized with different PFD1235w domains and plasma was obtained as described in the Methods section. Antibody reactivity to infected erythrocytes surface expressing PFD1235w was measured by flow cytometry and data given as MFI _sample_/MFI_sample prebleed_. The dotted line shows the cut-off defined as described in the Methods section.

**Figure 4:**
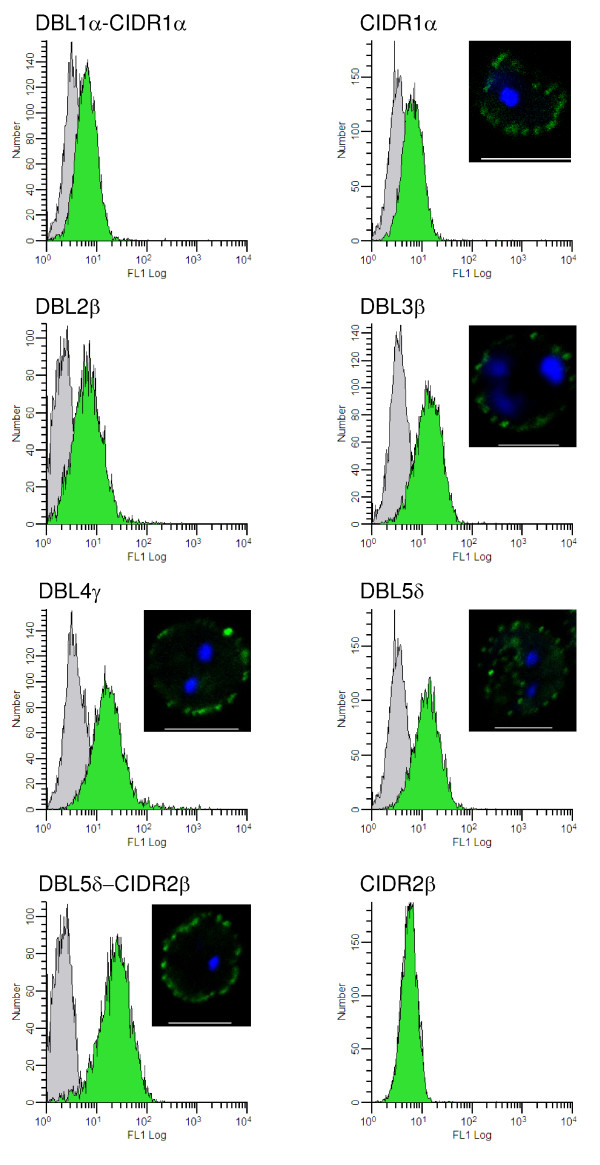
**IE surface reactivity of sera raised against insect cell produced domains of PFD1235w**. Shown is flow cytometry of PFD1235w expressing IE labelled with PFD1235w domains-specific rat antisera (green histograms) or with preimmunization control sera (grey histograms). The corresponding immunofluorescence microscopy surface reactivity is shown by inserts for antisera against CIDR1α, DBL3β, DBL4γ, DBL5δ and DBL5δ-CIDR2β. Preimmunization sera and antisera against VAR2CSA [37] were negative in both assays.

Flow cytometry was used to test the ability of the induced antisera to recognize native PFD1235w on the surface of erythrocytes infected with a 3D7_PFD1235w _parasite line selected for expression of high levels of this particular PfEMP1 [19]. Antisera raised against all the insect-cell produced domains were surface reactive with the 3D7_PFD1235w _line, except antisera to CIDR2β (Figure 1, Figure 3A, and Figure 4). In contrast, only antisera raised against DBL4γ produced in the *E. coli *Rosetta-gami B (DE3) strain contained IgG surface reactive with the surface of 3D7_PFD1235w _as did DBL1α-CIDR1α antisera although this protein did not express at detectable levels (Figure 1 and Figure 3A).

This demonstrates that the majority of the seven different domains are surface-exposed and accessible to antibodies on the surface of infected human erythrocytes that are expressing the PFD1235w PfEMP1 antigen (Figure 4). An increased reactivity to the surface of infected erythrocytes was observed with antisera targeting the C-terminal parts of PFD1235w as compared to sera targeting the N-terminal regions of this PfEMP1, irrespective of the production system (Figure 3B).

### Sharing of epitopes between insect cell and *E. coli *produced PFD1235w protein

**Figure 5:**
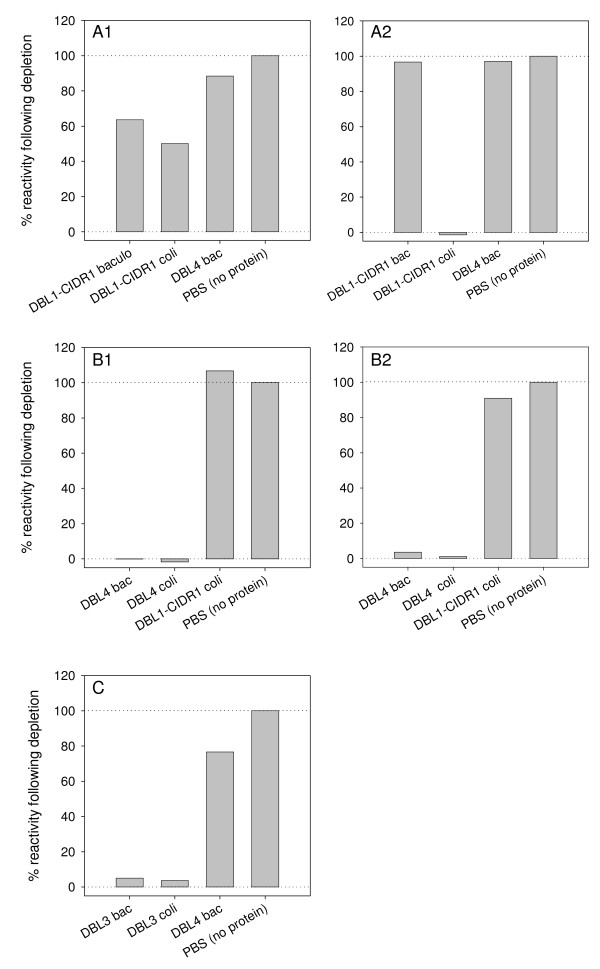
**Recognition of native PFD1235w expressed on the surface of 3D7_PFD1235w _IE**. The surface antibody reactivity of rat antisera raised against (A1 and B1) insect cells and (A2 and B2) *E. coli *produced DBL1α-CIDR1α and DBL4γ was measured by flow cytometry. (C) The antibody surface reactivity of rat antisera raised against insect cell produced DBL3β. Antisera were pre-incubated with excess homologous immunizing protein, heterologous produced protein, PBS or irrelevant protein as indicated. The percentage of reactivity left following depletion was calculated as described in the Methods section.

To compare the antibody epitope profile being exposed by insect cell and *E. coli *produced protein domains, rat antisera raised against the recombinant constructs was blocked by homologous as well as heterologous protein prior to flow cytometry testing (Figure 5). Both homologous and heterologous DBL4γ removed all IE surface antibody reactivity of both the heterologous and homologous anti-DBL4γ antisera (Figure 5B1 and 5B2) indicating that the two proteins are similarly folded and expose identical epitopes.

Similarly, *E. coli *produced DBL3β removed the surface antibody reactivity of the heterologous antisera, although this protein was unable to induce surface reactive antibodies in rats (Figure 5C). By contrast, the baculovirus produced DBL1α-CIDR1α was unable to block the reactivity of antisera raised against the heterologous protein. This indicates that there is a difference in the antibody epitopes being exposed in the rats following immunization (Figure 5A1 and 5A2). Doubling the concentration of blocking protein gave similar results. However, depletion using *E. coli *produced DBL1α-CIDR1α protein did lead to a reduction in the reactivity of the heterologous antisera (Figure 5A1).

### Antibodies acquired during *in vivo *infections recognize recombinant PFD1235w domains

**Figure 6:**
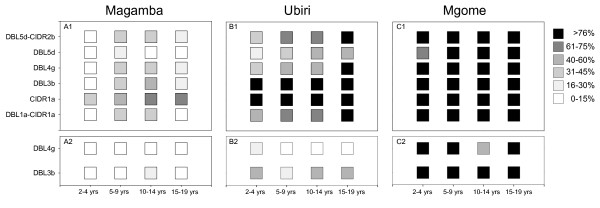
**Human antibody reactivity to PFD1235w**. Percentage antibody responders to recombinant PFD1235w domains produced in baculovirus-infected insect cells (A1-C1) and in *E. coli *(A2-C2). The antibody reactivity of plasma obtained from individuals living in three Tanzanian villages (A) Magamba, (B) Ubiri, and (C) Mgome of low, moderate, and high malaria transmission intensity was measured by Luminex. Each age group (2-4, 5-9, 10-14, and 15-19 years) included 15 individuals. The percentage responders was categorized into six different intervals: 0-15 (white), 16-30 (lighter gray), 31-45 (light gray), 46-60 (dark grey), 61-75 (darker grey), and >76 (black).

A bead-based Luminex assay [24] was used to assess antibody reactivity of plasma samples from 180 Tanzanian children with a previous history of malaria [29] and 20 Danish blood donors with no previous exposure to malaria (Figure 6).

The average mean fluorescent intensity (MFI) of the antibody reactivity of the Danish control plasma samples towards the six insect cell produced proteins (DBL1α-CIDR1α, CIDR1α, DBL3β, DBL4γ, DBL5δ, DBL5δ-CIDR2β) was low (MFI_average_= [29;80], MFI_min_= [0;5], MFI_max_=[84;349]) compared to the antibody reactivity to the bacterial produced DBL3β (MFI_average_= 151, MFI_min_= 6, MFI_max_= 525) and DBL4γ (MFI_average_= 351, MFI_min_= 4, MFI_max_= 1042) domains. This probably reflects naturally acquired antibodies reacting with host contaminants present in the batch of DBL3β and DBL4γ produced in the *E. coli *system. As a result of this reactivity the MFI cut-off value based on the reactivity in Danish plasma was higher for the *E. coli *proteins than for the insect cell produced PfEMP1 domains.

A relationship between transmission intensities, age groups and percentage of responders was observed for all eight recombinant PfEMP1 domains (Figure 6). The plasma antibody reactivity also increased with age and with increasing transmission intensity. However, the two bacterial produced proteins showed a lower percentage of positive responders compared to the homologous insect cell produced proteins. Of the 180 Tanzanian plasma samples 75% were either both positive or both negative in reactivity to insect cell and *E. coli *produced DBL3β and 65% both positive or both negative for DBL4γ. The number of plasma samples showing positive antibody reactivity to insect cell produced, but not *E. coli *produced protein was markedly higher than the opposite situation (DBL3β: 22% vs 3%, DBL4γ: 32% vs 2%).

## Discussion

Identification of heterologous expression systems capable of expressing milligrams of soluble recombinant *P. falciparum *protein is a challenging process. In this study, the use of *E. coli *and insect cells were compared in producing 44-93 kDa single- and double protein domains encoded by the *pfd1235w var *gene. All seven constructs introduced into the baculovirus expression system produced recombinant protein, while three of seven constructs produced protein in the prokaryotic system (Figure 1). The high molecular weight (≥ 57 kDa) of the four remaining *E. coli *constructs might explain why these did not express [4-6]. Decreasing the expression temperature to 4°C or varying concentrations of IPTG did not result in improved expression (data not shown).

*Plasmodium falciparum *proteins that are expressed in insoluble inclusion bodies in bacteria have previously been shown to be expressed in soluble form and capable of being made and purified in milligram amounts upon transfer to the baculovirus system [4]. However, the baculovirus expression system has yet to prove its worth when it comes to crystallography of PfEMP1 proteins, since five out of the six published crystal structures to date have been done on bacterially expressed recombinant proteins [30-35]. In addition, insect cells are known to sometimes hyper-glycosylate their recombinant products, a potential problem for expression of PfEMP1, as N- and O-glycosylations seem to occur at very low levels in *P. falciparum *[36].

The insect cell produced proteins were between 60-97% pure as compared to 3-95% for the *E. coli *proteins. This difference can probably be ascribed to the fact that the insect cell expressed proteins are purified from the cell media, as opposed to the bacterially expressed protein, which were purified from total cell extract. Degradation and co-purification of degradation products was observed with both expression systems, but was especially evident in the *E. coli *expression system. In addition, larger bands were visible in non-reducing lanes on Western blots for all the expressed recombinant proteins, possibly representing dimers and trimers.

Flow cytometry was used to test the ability of antisera to recognize native PFD1235w on the surface of erythrocytes infected with 3D7_PFD1235w _a parasite line selected for high surface expression of PFD1235w [19]. Antisera raised against all of the seven baculovirus proteins and against PFD1235w DBL4γ and DBL1α-CIDR1α domains produced in the Rosetta-gami B (DE3) *E. coli *strain showed IgG reactivity with the surface 3D7_PFD1235w. _In contrast to a previous study [37], this also showed the ability of the *E. coli *expression system to produce PfEMP1 protein domains with a three-dimensional structure sufficiently resembling that of the native molecule to induce surface reactive antibodies following experimental immunization.

The insect cell produced DBL4γ consistently induce surface reactive IgG in rats, whereas this is not always observed with recombinant DBL4γ expressed by *E. coli*. This would indicate the *E. coli *expression system to be more sensitive to batch to batch variation.

To assess similarities in exposed antibody epitopes homologous and heterologous antisera were pre-incubated with recombinant DBL4γ, DBL3β and DBL1α-CIDR1α produced in both expression systems prior to testing by flow cytometry (Figure 5). The two DBL4γ domains exposed identical epitopes as the reactivity of the antisera raised against insect cell produced DBL4γ could be abolished by pre-incubating with *E. coli *produced DBL4γ and vice versa (Figure 5B1 and 5B2). Similarly, DBL3β produced in *E. coli *abolished the reactivity of the heterologous antisera following depletion although this protein did not induce surface reactive antibodies following immunization of rats. However, this was not the case for the DBL1α-CIDR1α antisera indicating that the insect and bacterial derived proteins were fundamentally different due to differences in folding and/or post-translational modifications. These results show that most PFD1235w domains are surface exposed and accessible to antibodies in the native molecule as expressed by IE and that induction of surface reactive IgG seem critically dependent on a correct three-dimensional structure of the antigen used for immunization, a structure which is more often achieved in the eukaryotic compared to the prokaryotic system used. Depletion of DBL1α-CIDR1α and DBL5δ-CIDR2β antisera using either CIDR1α, DBL5δ or CIDR2β protein produced in insect cells show a reduced reactivity by FACS. This strongly indicates DBL1α and CIDR2β to be similarly exposed on the native PFD1235w exported to the surface of IE.

Six of the seven insect cell and two of the *E. coli *produced proteins were used in a bead based Luminex assay to assess the antibody reactivity of 180 plasma samples collected from individuals with a previous exposure of malaria and living in a high (Mgome), moderate (Ubiri), and low (Magamba) malaria transmission area of Tanzania [23]. The MFI_cut-off _value for the bacterial derived proteins in the Luminex analysis was considerable higher than their homologous counterparts from the baculovirus expression system. This cannot be explained by reactivity against the V5 tag as all proteins irrespective of expression system carry this, but is more likely a consequence of the low purity of the bacterial recombinant proteins samples.

Although the *E. coli *produced DBL3β did not induce any surface reactive antibodies in rats both domains were recognized by African sera in the Luminex assay. However, at a reduced level as compared to the equivalent insect cell produced protein (Figure 6). All proteins irrespective of production system were recognized by plasma antibodies in an age and transmission dependent manner. This correlation between malaria transmission intensity, age and acquisition of antibodies is in line with our previous finding on antibody responses to PFD1235w [20].

## Conclusions

This study, assessed the use of a eukaryotic and prokaryotic system for expression of PFD1235w protein and found the insect cells more reliable and superior to *E. coli *in producing recombinant PfEMP1 domains that generates IE surface reactive antibodies following immunization of rats.

## Abbreviations

IE: infected erythrocyte; ICAM1: intracellular adhesion molecule 1; MSP1: merozoite surface protein 1; PfEMP1: *Plasmodium falciparum *erythrocyte membrane protein 1; NTS: N-terminal segment; DBL: Duffy-binding like domain; CIDR: cysteine-rich interdomain;

## Competing interests

The authors declare that they have no competing interests.

## Authors' contributions

MEV, AB, GA, DB, LJ, ATRJ designed the study and performed laboratory work, JPL, LSV and TGT organized the field work. ATRJ and MEV wrote the manuscript. AB, GA, DEA, LJ, TT helped to draft the manuscript. All authors have read and approved the final manuscript and contributed significantly to this work.

## Supplementary Material

Additional file 1 **Table 1. Primers used for amplification of DNA encoding PFD1235w domains**. The table provides information on primers used for PCR amplification of amplicons encoding different PFD1235w protein domains. Click here for file
