# Supplementary material for: Insect cells are superior to Escherichia coli in producing malaria proteins inducing IgG targeting PfEMP1 on infected erythrocytes
Source: Malar J. 2010 Nov 15;9:325. doi: 10.1186/1475-2875-9-325 (PMC2994891; doi:10.1186/1475-2875-9-325)
Supplement: Additional file 1 — Table 1. Primers used for amplification of DNA encoding PFD1235w domains. The table provides information on primers used for PCR amplification of amplicons encoding different PFD1235w protein domains. [file 1475-2875-9-325-S1.DOCX]

# Additional files

### Table 1. Primers used for amplification of DNA encoding PFD1235w domains.

|  | Domain | Construct | Primer # |
| --- | --- | --- | --- |
| pAcGP67A transfer vector^c^ | NTS-CIDR1a ^a^* | 1359c | 5´-GAATTCATGGGGAATGCATCATCA-3´  5´-ATAAGAAT**GCGGCcgc**TACAGGGGTTTGTTTTCGTATC-3´ |
|  | DBL1α-CIDR1α^b^ | 1292C | 5'-CACCATG*CCCGGG*TGCGAGCTGGACTACCGCTTC-3'  5'-GAATTCGCAAGGGTTGGTCTTGGTGTCCACGG-3' |
|  | CIDR1α | 1199C | 5'-CGGAATTCGACTATTGCCAAATATGTC-3'  5'-ATAAGAAT**GCGGCCGC**TACCTGTACATGGTTCACC-3' |
|  | DBL2β | 1357C | 5'-CACCATGGAATTCACCGGTTGTGCTAAACCTCCTGGTAGTAAAC-3'  5'-**GCGGCCGC**TCCTAGGACACTTACACGCCTCAACATATAC-3' |
|  | DBL3β | 1358C | 5'-CACCATGGAATTCACCGGTTGTGCTGAAACTGGTGGTGTACATACC-3'  5'-**GCGGCCGC**TCCTAGGGCAATCACACGCTGTAGCATAC-3' |
|  | DBL4γ | 1334C | 5'-GGAATTCTGCAAAATAGTGGAAGAAG-3'  5'-ATAAGAAT**GCGGCCGC**GACAATTACACTTTCCAT-3' |
|  | DBL5δ-CIDR2β | 1361C | 5'-CACCATG**CAATTG**ACCGGTTGCGCCACAGTGGCCAAAG  5'-**GCGGCCGC**TCCTAGGACAAATGTTCGGTTGTGTTACTGGG |
|  | DBL5δ | 1259C | 5'-CGGAATTCGCATGTGCCCTCAAATATG-3'  5'-ATAAGAAT**GCGGCCGC**GGCAATCCTTTTGCTTAGCAC-3' |
|  | CIDR2b* | 1398C | 5´-AT*CCCGGG*TGTGAAAATGGTAGTTGTGGGAGTG-3´  5´-AT**GCGGCCGC**TACACTTTTCTTCTTCCTC-3´ |
|  | DBL1α-CIDR1α | 1220C | 5'-CACCATGCCCGGGTGCGAGCTGGACTACCGCTTC-3'  5'-GAATTCGCAAGGGTTGGTCTTGGTGTCCACGG-3' |
|  | CIDR1α | 1387C | 5'-CACCATGGCGGATCCCGGG-3'  5'-TCCCAGGAAAGGATCAGATCTGC-3' |
| pET101/D-TOPO vector^d^ | DBL2β | 1388C | 5'-CACCATGGCGGATCCCGGG-3'  5'-TCCCAGGAAAGGATCAGATCTGC-3' |
|  | DBL3β | 1365C | 5'-CACCATGGAATTCACCGGTTGTGCTGAAACTGGTGGTGTACATACC-3'  5'-GCGGCCGCTCCTAGGGCAATCACACGCTGTAGCATAC-3' |
|  | DBL1α-CIDR1α | 1353C | 5'-CACCATGGCGGATCCCGGG-3'  5'-TCCCAGGAAAGGATCAGATCTGC-3' |
|  | DBL5δ-CIDR2β | 1368C | 5'-CACCATGGCGGATCCCGGG-3'  5'-TCCCAGGAAAGGATCAGATCTGC-3' |
|  | DBL5δ | 1385C | 5'-CACCATGGCGGATCCCGGG-3'  5'-TCCCAGGAAAGGATCAGATCTGC-3' |

^a^Domain cloned into the pBAD-TOPO vector (Invitrogen). For production of carboxy terminally V5 epitope and histidine-tagged protein the domain was excised by EcoRI and PmeI digestion and then subcloned into the EcoRI and blunt-ended BglII sites of pAcGP67-A. *Domains only produced in insect cells. ^b^The sequence was re-codonised for expression in the baculovirus expression system. ^c^Restriction enzyme sites: EcoRI underlined, NotI in bold, XmaI underlined and in italics, MfeI bold and underlined. ^d^All inserts were directional blund-end cloned into the pET101/D-TOPO vector.
